# Post-COVID-19 Inflammatory Myopathy With Dermatomyositis–Mixed Connective Tissue Disease (MCTD) Overlap Features and Epiglottitis: A Case Report

**DOI:** 10.7759/cureus.99362

**Published:** 2025-12-16

**Authors:** Nicholas Lorenz, Amia Mourad, Grant Weiderman, Juan Daniel Pulido

**Affiliations:** 1 Orthopaedic Surgery, Lake Erie College of Osteopathic Medicine (LECOM) - Bradenton, Bradenton, USA; 2 General Medicine, Lake Erie College of Osteopathic Medicine, Bradenton, USA; 3 Anesthesiology, Lake Erie College of Osteopathic Medicine, Bradenton, USA; 4 Critical Care, Baptist Medical Center Beaches, Jacksonville, USA

**Keywords:** dermatomyositis, epiglottitis, inflammatory myopathy, mixed connective tissue disease, post-covid-19 autoimmunity, post-covid-19 complications

## Abstract

Autoimmune inflammatory myopathies can emerge after viral illnesses and may present with a wide range of neuromuscular and systemic symptoms. Post-viral immune dysregulation may unmask or trigger overlap syndromes characterized by muscle inflammation, dysphagia, airway involvement, and broader immune activation. This report describes a case of inflammatory myopathy with dermatomyositis and mixed connective tissue disease-associated antibodies occurring after a recent COVID-19 infection, highlighting the diagnostic challenges and severity of presentation. The case underscores the importance of recognizing post-COVID autoimmune complications and initiating timely immunosuppressive therapy to reduce long-term morbidity.

## Introduction

Viral infections are known to trigger a range of post-infectious inflammatory and autoimmune phenomena involving the neuromuscular, rheumatologic, and upper aerodigestive systems. Although traditionally uncommon, supraglottitis and epiglottic inflammation have emerged as increasingly recognized but still rare manifestations associated with respiratory viruses, including COVID-19 [1]. Proposed mechanisms include direct viral involvement of the upper airway mucosa, expression of angiotensin-converting enzyme 2 (ACE2) receptors within the epiglottic and supraglottic tissues, and amplified innate immune signaling leading to localized edema and mucosal irritation [2]. These processes can produce significant airway inflammation and dysphagia, sometimes persisting even after the acute viral phase has resolved.

While myalgias are common during viral illnesses, true autoimmune inflammatory myositis is a far less frequent post-infectious complication, even in the setting of COVID-19. Dermatomyositis (DM) is an inflammatory myopathy characterized by proximal muscle weakness, characteristic cutaneous findings, and immune-mediated perifascicular muscle fiber injury [3]. Mixed connective tissue disease (MCTD) is defined by high-titer anti-U1-RNP antibodies and features overlapping systemic lupus erythematosus, scleroderma, and polymyositis [4]. Overlap syndromes occur when elements of multiple autoimmune diseases coexist, driven by shared immune pathways [3].

Post-infectious onset of these conditions can occur through several underlying immunologic mechanisms. Viral antigens can resemble skeletal muscle proteins, leading to molecular mimicry and the activation of autoreactive lymphocytes [5]. Persistent type I interferon signaling, strongly associated with dermatomyositis, can promote perifascicular fiber injury, MHC class I upregulation, and an inflammatory cascade directed at muscle tissue. In addition, endothelial and microvascular injury caused by viral infection may trigger complement activation, ischemia, and capillary dropout, further amplifying muscle damage. Dysregulated T-cell responses, including expansion of CD4+ and cytotoxic lymphocyte subsets, can perpetuate immune-mediated myofiber necrosis [6].

COVID-19 appears to engage many of these same pathways, which likely accounts for its emerging association with post-infectious myositis [7]. However, autoimmune inflammatory myopathies may also develop from other triggers, including malignancy, certain medications, environmental exposures, and other viral infections [8]. In contrast to these more established etiologies, COVID-19-associated inflammatory myositis remains less common but increasingly recognized.

This case illustrates a patient who developed severe supraglottic inflammation, profound proximal muscle weakness, dysphagia, and serologic evidence of a dermatomyositis with MCTD overlap syndrome following recent COVID-19 infection. Her presentation demonstrates the potential for a complex, multisystem autoimmune response affecting both the upper airway and skeletal muscle, representing a very unique clinical pattern.

## Case presentation

**Figure 1:**
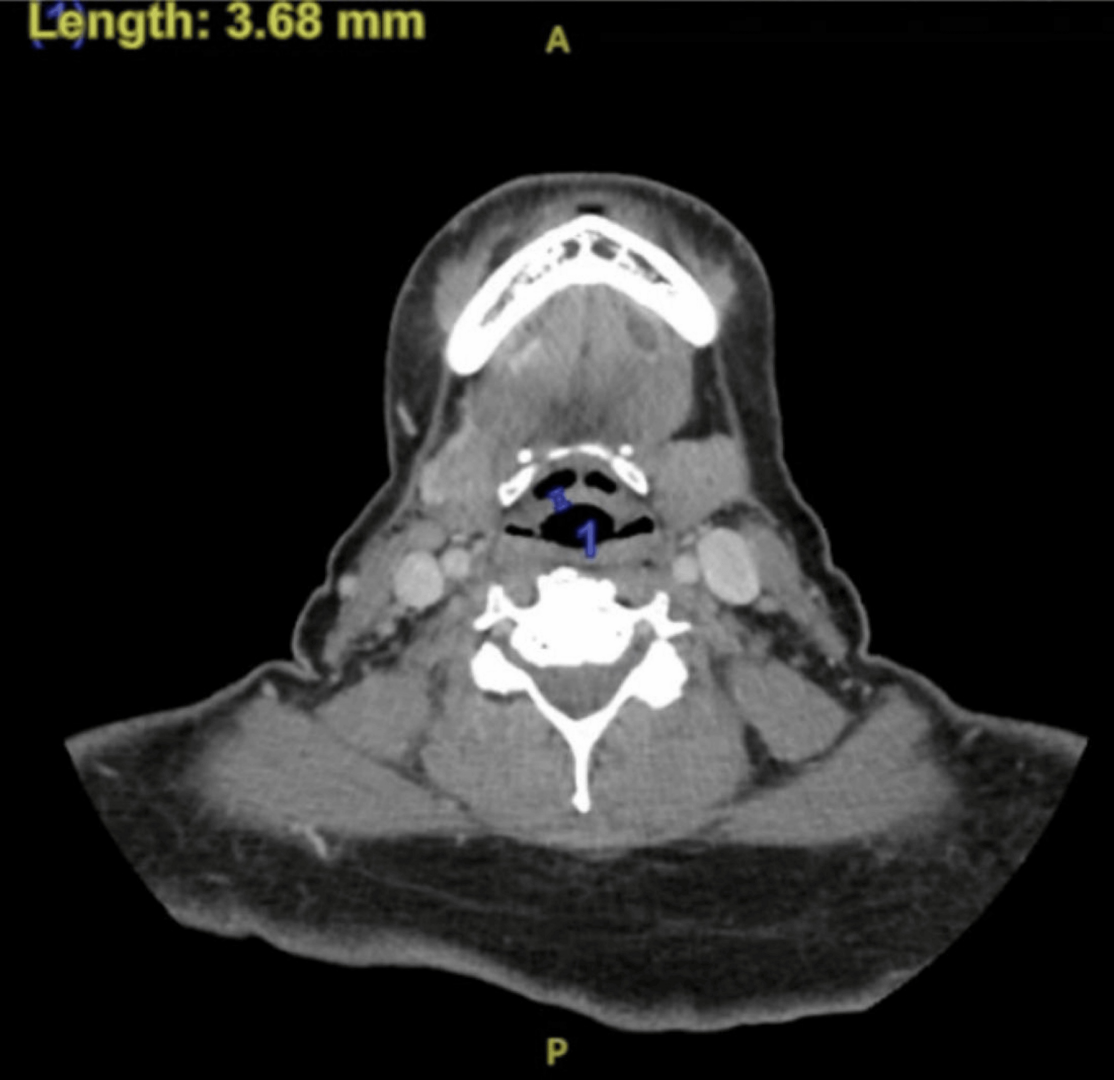
Axial CT of the Infrahyoid Neck Showing Mild Diffuse Epiglottic Thickening Measuring 3.68 mm, Consistent with Supraglottitis

A 62-year-old woman with a history of obesity presented with progressively worsening dysphagia, throat discomfort, vomiting with solid foods, and increasing difficulty rising from a seated position. She had recently recovered from COVID-19 one month ago and reported persistent anorexia and pharyngeal discomfort since that time. On examination, she was hypertensive and tachycardic, with marked pharyngeal edema, mucosal erythema, and exudates. Initial laboratory testing demonstrated elevated aspartate aminotransferase (AST 324 U/L), alanine transaminase (ALT 157 U/L), erythrocyte sedimentation rate (ESR 56 mm/hour), C-reactive protein (CRP 3.5 mg/dL), lactate dehydrogenase (LDH 647 U/L), and increased troponin I (149 pg/mL) (Table 1). CT imaging of the neck revealed diffuse epiglottic thickening consistent with supraglottitis, prompting admission for airway monitoring and initiation of corticosteroids, antimicrobial therapy, IV fluids, and electrolyte repletion (Figure 1).

**Table 1:** Key Laboratory Values Over the Course of Illness CK: creatinine kinase; ESR: erythrocyte sedimentation rate; CRP: c-reactive protein; LDH: lactate dehydrogenase; AST: aspartate aminotransferase; ALT: alanine transaminase

	Reference Range	Admission	Discharge	Follow-Up
Total CK	38 - 234 U/L	2847	619	157
ESR	0.0 - 30.0 mm/hour	56.0	-	9
CRP	0.5 - 1.0 mg/dL	3.5	-	5
LDH	100 - 190 U/L	647	-	-
AST	15 - 41 U/L	324	52	27
ALT	14 - 54 U/L	157	48	30
Myoglobin	<=66 mcg/L	2060	-	-
Aldolase	<=8.1 U/L	20.8	-	-
Troponin I	<12 PG/ML	149	-	-

Shortly after admission, she failed a swallow evaluation and required nasoenteric tube placement. Around the same time, she developed rapidly progressive, symmetric proximal muscle weakness, most pronounced in the upper extremities, with deltoid strength declining to approximately 1/5 bilaterally. Lower extremity strength, initially preserved, also began to weaken. Laboratory testing obtained during this progression revealed a creatine kinase level of 2847 U/L and an aldolase of 20.8 U/L, accompanied by significant myoglobinuria, raising concern for an inflammatory myopathy with associated rhabdomyolysis (Table 1). Neurologic evaluation was pursued, and cerebrospinal fluid analysis did not support Guillain-Barré syndrome.

**Figure 2:**
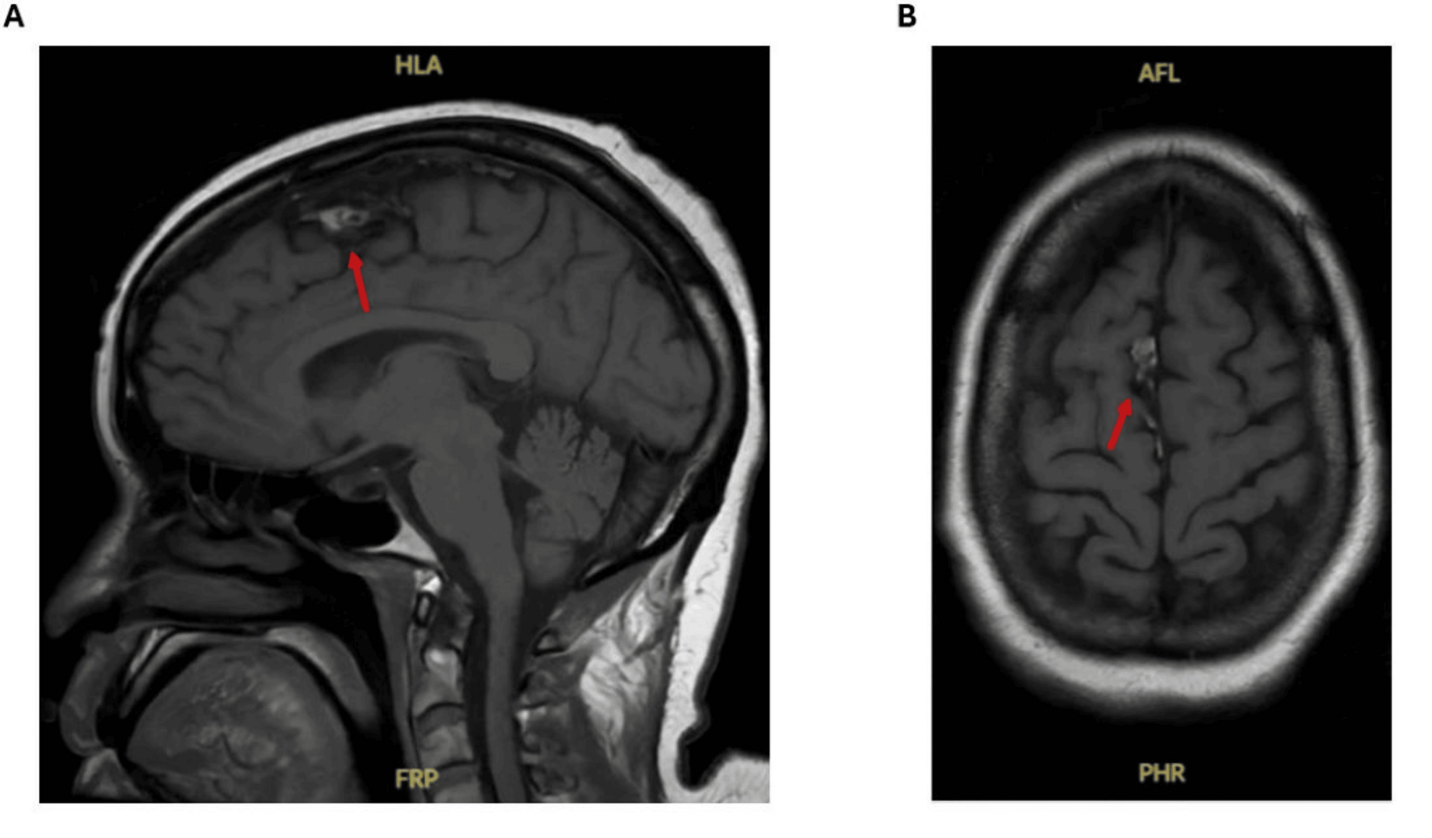
Sagittal (A) and Axial (B) MRI Sequences Showing a Punctate Acute Infarct in the Right Precentral Gyrus

MRI of the brain demonstrated a punctate acute infarct in the right precentral gyrus; however, the lesion’s size and focal cortical distribution did not explain her profound, symmetric proximal weakness or bulbar symptoms (Figure 2). The clinical pattern was inconsistent with a cortical process and strongly suggested a primary myopathic disorder. Her dysphagia progressed despite improvement in supraglottic swelling, eventually requiring placement of a percutaneous endoscopic gastrostomy tube for medication administration and nutrition.

**Figure 3:**
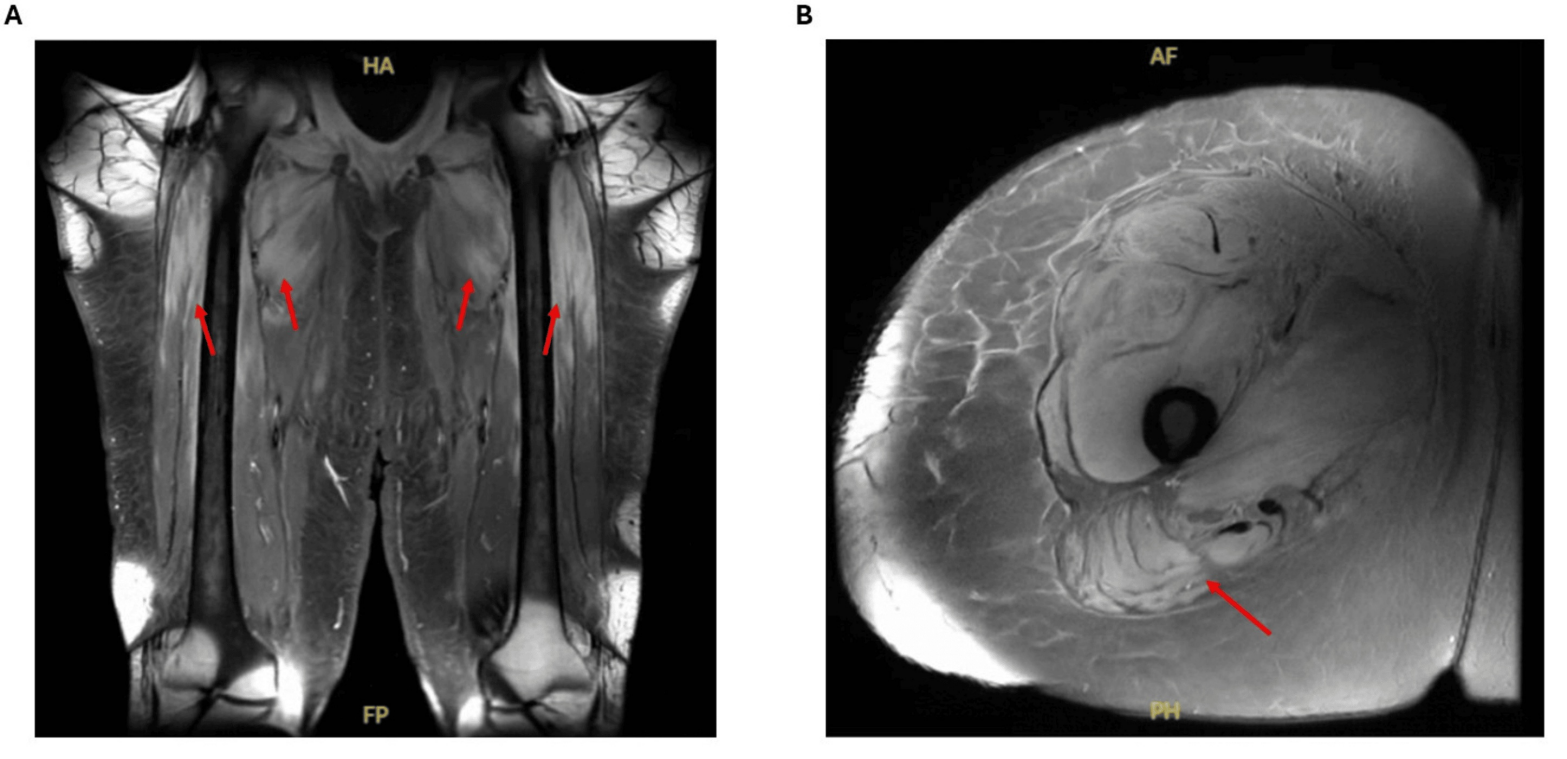
Coronal (A) and Axial (B) MRI Sequences Showing Diffuse Muscle Edema Involving the Anterior, Posterior, and Medial Compartments of the Proximal Thighs, Consistent with Active Inflammatory Myositis

Further diagnostic evaluation revealed additional systemic involvement. MRI of the right femur showed extensive T2 hyperintensity and contrast enhancement throughout the anterior, medial, and posterior thigh compartments, consistent with active inflammatory myositis (Figure 3). Transthoracic echocardiography with bubble study demonstrated delayed appearance of microbubbles consistent with intrapulmonary shunting. CT angiography confirmed pulmonary arteriovenous malformations, and troponin I remained elevated, supporting myocardial involvement. MRA of the neck revealed moderate-to-severe vertebral artery stenosis without evidence of an acute occlusive event.

Autoimmune serologic testing demonstrated a high-titer antinuclear antibody (ANA) of ≥1:1280 with a speckled pattern, strongly positive U1-ribonucleoprotein antibody (U1-RNP) at 18.0 U/mL, and markedly elevated Mi-2α and Mi-2β antibodies measuring 110 SI and 34 SI, respectively. Antibodies to double-stranded DNA (dsDNA), Smith (Sm), Sjogren’s syndrome A and B (SSA/SSB), histidyl-tRNA synthetase (Jo-1), topoisomerase I (Scl-70), and other myositis-specific autoantibodies were negative (Table 2). A muscle biopsy of the thigh revealed perifascicular atrophy, necrotic and regenerating fibers, endomysial and perivascular inflammation, and a CD4-predominant lymphocytic infiltrate without rimmed vacuoles or inclusion bodies, confirming an autoimmune inflammatory myopathy with dermatomyositis-spectrum and connective tissue overlap features.

**Table 2:** Autoimmune and Myositis-Specific Antibody Panel Results ANA: antinuclear antibody; dsDNA: double-stranded DNA antibody; Sm: smith antibody; U1-RNP: U1-ribonucleoprotein antibody; SSA/Ro: Sjogren’s syndrome A antibody; SSB/La: Sjogren’s syndrome B antibody; Jo-1: histidyl-tRNA synthetase antibody; Scl-70: topoisomerase I antibody; ANCA: antineutrophil cytoplasmic antibody

	Reference Range	Patient Value
ANA	Negative	Positive
ANA Titer	<1:40	>=1:1280
dsDNA Antibody	<=14.9 U/mL	1.4
Sm	<=4.9	0.9
U1-RNP	<=4.9 U/mL	18.0
SSA/Ro	<=6.9 U/mL	0.4
SSB/La	<=6.9 U/mL	0.4
Jo1 Antibody		0.3
Centromere Antibody	<=6.9 U/mL	0.5
Anti-SCL70	<=6.9 U/mL	1.2
ANCA	Negative	Negative
Mi-2 Alpha Ab	<11 SI	110
Mi-2 Beta Ab	<11 SI	34

Given the severity of her inflammatory myopathy, the patient received three days of pulse-dose IV methylprednisolone, followed by transition to high-dose daily prednisone delivered through the PEG tube. Rheumatology initiated weekly methotrexate with folic acid supplementation as steroid-sparing therapy. She was started on atovaquone for *Pneumocystis jirovecii* pneumonia prophylaxis due to prolonged high-dose steroid exposure. Additional management included aggressive IV hydration for rhabdomyolysis, electrolyte repletion, anticoagulation for venous thromboembolism (VTE) prophylaxis, and intensive physical and occupational therapy. Her dysphagia and nutritional deficits were managed through the PEG tube.

Serum values improved significantly with treatment. By discharge, CK had decreased to 619 U/L, AST to 52 U/L, and ALT to 48 U/L (Table 1). She was discharged to acute rehabilitation on high-dose prednisone, weekly methotrexate, folic acid, and atovaquone prophylaxis. At outpatient rheumatology follow-up, she demonstrated meaningful functional recovery, with improved swallowing, partial return of proximal strength, and the ability to ambulate with assistance. Her laboratory evaluation continued to show improvement, with CK falling within range at 157 U/L and ESR reduced to 9 mm/h (Table 1), consistent with decreasing inflammatory activity while maintained on immunosuppressive therapy.

## Discussion

This patient developed a severe autoimmune inflammatory myopathy with prominent proximal weakness, bulbar dysfunction, and early supraglottic involvement. Although supraglottic edema initially raised concern for a primary airway process, its rapid improvement contrasted with the continued progression of her profound weakness and dysphagia. Rather than serving as the primary cause of her symptoms, the supraglottic inflammation appeared to be another manifestation of a broader post-infectious inflammatory response. The small acute cortical infarct seen on MRI brain likewise could not account for the symmetric proximal weakness or the severity of her swallowing impairment. The overall pattern of weakness, MRI muscle edema, markedly elevated CK, elevated AST and ALT, paralleling muscle injury, and muscle biopsy findings were all consistent with an inflammatory myopathy rather than a central neurologic or isolated airway disorder.

Her autoimmune profile, showing high-titer ANA, strongly positive U1-RNP, and markedly elevated Mi-2α and Mi-2β antibodies, supported a dermatomyositis-spectrum inflammatory myopathy with connective tissue overlap features [9]. Mi-2 antibodies target components of the nucleosome remodeling-deacetylase (NuRD) complex involved in chromatin remodeling, contributing to perifascicular fiber vulnerability typical of dermatomyositis [10]. U1-RNP antibodies target the U1 small nuclear ribonucleoprotein complex involved in mRNA splicing and are characteristic of mixed connective tissue disease [11]. Despite having a dermatomyositis-associated autoantibody profile, she exhibited no classic cutaneous manifestations. This occurs in a subset of patients with clinically amyopathic or hypomyopathic dermatomyositis, in whom the immunologic injury predominantly targets muscle or internal organs rather than the skin [12]. Furthermore, classic MCTD often includes Raynaud phenomenon, arthritis, and features of lupus or scleroderma, none of which were present in this patient [13]. This atypical clinical pattern, combined with her distinctive antibody profile, suggests broad activation of multiple nuclear antigen pathways consistent with multisystem involvement.

She had no alternative triggers, including no new medications, no statin exposure, no evidence of malignancy, and no metabolic or endocrine abnormalities that might explain inflammatory muscle injury. Her symptoms began within the well-recognized 2-6-week post-infectious interval during which autoimmune phenomena commonly surface [14]. Her elevated AST and ALT improved in parallel with CK and clinical recovery, supporting a muscle-derived source rather than primary hepatic injury.

The pathophysiology of post-infectious inflammatory myositis likely reflects a convergence of immune pathways directed specifically at skeletal muscle. One proposed mechanism involves loss of self-tolerance, wherein viral immune activation exposes normally sequestered nuclear or cytoplasmic muscle antigens, allowing autoreactive T and B cells to expand [5]. In this setting, autoantibody formation against targets such as Mi-2 and U1-RNP may emerge as part of a broader nuclear antigen response, contributing to both muscle fiber injury and systemic overlap features [15,16]. Aberrant activation of interferon-regulated genes within muscle tissue can further exaggerate immune-mediated fiber damage, promoting perifascicular atrophy and sustained inflammatory signaling. Additionally, microvascular dysfunction within muscle, including capillary dropout and complement deposition, may reduce perfusion and exacerbate injury [6]. Together, these processes offer a mechanistic explanation for the patient’s biopsy findings and the severity of her clinical presentation.

Although post-viral myositis has been described, this case was distinguished by its multisystem involvement, including severe bulbar dysfunction requiring PEG placement and laboratory evidence of myocardial injury. The coexistence of Mi-2 and U1-RNP positivity further reflects a broad autoimmune overlap rather than a single-pathway myopathy. Her significant improvement with corticosteroids and methotrexate supports an immune-mediated etiology.

Overall, the timing of symptom onset, absence of alternative triggers, serologic pattern, airway involvement, and biopsy findings strongly support a post-infectious autoimmune inflammatory myopathy as the underlying cause of her presentation.

## Conclusions

This case demonstrates a severe inflammatory myopathy with dermatomyositis-spectrum and connective tissue overlap features occurring after recent COVID-19 infection. The presentation included profound symmetric proximal weakness, dysphagia, supraglottic inflammation, and multisystem involvement. Diagnostic evaluation revealed markedly elevated muscle enzymes, MRI evidence of myositis, autoantibody positivity including Mi-2 and U1-RNP, and biopsy-confirmed inflammatory muscle injury. Early initiation of corticosteroids and methotrexate resulted in significant clinical and biochemical improvement. This case highlights the importance of recognizing post-COVID autoimmune myopathies, particularly when patients present with new proximal weakness or dysphagia following infection.
